# COGNITIVE APPRAISAL AND KNOWLEDGE AS RISK FACTORS FOR HIGH FEAR OF FALLING IN OLDER AND MIDDLE-AGED ADULTS IN SINGAPORE

**DOI:** 10.1093/geroni/igz038.1762

**Published:** 2019-11-08

**Authors:** Pey June Tan, Reuben Ng, Angelique Chan, Jagadish U Mallya, Noor Hafizah Ismail, Chek Hooi Wong, Peter K Tay

**Affiliations:** 1 Geriatric Education and Research Institute, Singapore, Singapore; 2 Centre for Ageing Research and Education, Duke-NUS Medical School, Singapore, Singapore; 3 Department of Geriatric Medicine, Khoo Teck Puat Hospital, Singapore, Singapore; 4 Department of Continuing and Community Care, Tan Tock Seng Hospital, Singapore, Singapore; 5 Centre for Ageing Research and Education, Singapore, Singapore

## Abstract

Fear-of-falling (FOF) can be adaptive or maladaptive depending on one’s appraisal of knowledge and beliefs, but few have elucidated this cognitive process in older adults surrounding falls. We aim to identify risk factors for high FOF amongst community-dwelling older adults (OA) and middle-aged adults (MA) in Singapore. This was a cross-sectional survey of a nationally-representative sample of OA (≥60 years) and MA (40-59 years) identified by stratified random sampling. Primary outcome was high FOF measured by a single-item (4-point scale). Independent variables were history-of-falls, quality-of-life, fall-related cognitive appraisal (balance problems, importance to restrict activities to prevent falls) and knowledge indicators (knowledge of other OA who fell, ability to identify out of 13 fall risk factors). MA were also asked if they’re caregivers. Multiple logistic regressions identified risk factors for high FOF separately by age-groups, adjusting for socio-demographics and comorbidities. The final analysis included 549 OA (70.6±6.88 years) and 309 MA (49.7±5.89 years). No differences in high FOF was found among OA and MA (37% vs. 38%, p=0.305), but there were more falls among OA (19% vs 12%, p=0.010). Higher knowledge of fall risk factors and self-reported balance problems were significant risk factors for high FOF among OA only, while a history-of-falls and being a caregiver were significant among MA only. Perceived importance to restrict activities was associated with high FOF in both age-groups. Although findings suggest differences in the mechanism of high FOF between OA and MA, both age-groups have maladaptive appraisal tendencies related to restrict activities to prevent falls.

